# Hyperbilirubinemia Influences Sleep-Wake Cycles of Term Newborns in a Non-Linear Manner

**DOI:** 10.1371/journal.pone.0169783

**Published:** 2017-01-10

**Authors:** Lian Zhang, Yanxia Zhou, Xufang Li, Tingting Cheng

**Affiliations:** 1 Department of Neonatology, Guangzhou Women and Children’s Medical Center, Guangzhou, P. R. China; 2 Department of Neurology, Guangzhou Women and Children’s Medical Center, Guangzhou, P. R. China; Laval University, CANADA

## Abstract

Hyperbilirubinemia is a common cause for irreversible neuronal influence in the brain of term newborns, while the feature of neurological symptoms associated with hyperbilirubinemia has not been well characterized yet. In the present study, we examined a total of 203 neonates suffering from hyperbilirubinemia with a bedside amplitude-integrated Electroencephalography (aEEG) device, in order to determine whether there is any special change in sleep-wake cycles (SWCs). Among these patients, 14 cases showed no recognizable SWCs with the total serum bilirubin (TSB) level at 483.9–996.2 μmol/L; 75 cases exhibited reduced SWCs with the TSB level at 311.2–688.5 μmol/L; and the rest cases had the normal SWCs. The number of the normal SWCs occurrence had a significant negative correlation with the increased TSB level in a non-linear manner (*r = -0*.*689*, *p <0*.*001*). In addition, the increased TSB reshaped the structure of SWC by narrowing down the broadband and broadening the narrowband. Spearman’s correlation analysis indicated a significant negative correlation between the TSB level and the ratio of broadband (*r = -0*.*618*, *p < 0*.*001*), a significant positive correlation between the TSB level and the narrowband ratio (*r = 0*.*618*, *p < 0*.*001*), respectively. Furthermore, the change of SWC seemed like a continuous phenomenon, and the hyperbilirubinemia caused SWC changes was fit into a loess model in this paper. In summary, the hyperbilirubinemia influenced SWC of term newborns significantly at a non-linear manner, and these results revealed the feature of the neurological sequela that is associated with TSB.

## Introduction

Phototherapy and blood exchange transfusion progress, the incidence and mortality of kernicterus among full-term infants have been significantly reduced worldwide. In recent years, some of the more hidden or mild brain injury is defined as "bilirubin-induced neurological dysfunction" (BIND)[1,2]. BIND babies in the neonatal period may not have the typical signs of bilirubin encephalopathy, but BIND dose affect babies’ long-term intelligence, hearing, speaking, language and visual motor development[1]. Our previous study on animal models confirmed that bilirubin at low level does not affect the normal synaptic transmission in the hippocampus, but inhibited the generation of long term potentiation, leading to neuroplasticity changes[3]. This action could continue even after the bilirubin was removed from brain tissues. In clinical observations, such minor nerve damage is difficult to find through conventional means of inspection. However, some methods have been applied clinically to observe BIND to some extent.

When hyperbilirubinemia is not serious, brain MRI abnormalities are minor and can be easily overlooked[4]. Although brainstem auditory evoked potentials (BAEP) plays a certain role in early diagnosis of acute bilirubin encephalopathy, the most sensitive region of the auditory system is in the brainstem auditory nucleus[2], and the brainstem toxicity of bilirubin is not just in the auditory pathway [5]. Some studies recommended detection of bilirubin / albumin (B / A) ratio during hyperbilirubinemia [6], but there is no significant correlation between B / A ratio and bilirubin-induced acute or chronic neurological dysfunction[7]. Gürses et al [8] applied EEG and observed dysplasia of EEG activity main frequency in infants with hyperbilirubinemia. The results confirmed the inhibitory effect of hyperbilirubinemia on functions of central nervous system, but the researchers did not observe the sleep maturity of neonatal period. Given the limitations of the above diagnostic measures, in order to develop appropriate treatment strategies and to improve the nerve prognosis of neonatal jaundice, it is necessary to conduct careful neurological monitoring for BIND patients. Since the continuous dynamic monitor of TSB is not possible at present, the BIND signs are always analyzed with the TSB levels retrospectively. In fact these concerns could not be treated as a true representation of the causal relationship on the bilirubin exposure. However, a convenient tool to predict hyperbilirubinemia associated dysfunction at a given TSB level lacks.

Sleep is an important neural activity in the neonatal period, especially complete sleep, which is essential for the developing brain [9]. One sign of maturation of the nervous system is a complete sleep—wake cycle (SWC). SWC or SWC disorder is a serious abnormal state. The application of amplitude-integrated electroencephalography (aEEG) in the neonatal intensive care unit for bedside real-time monitoring can provide information on newborn SWC. The appearance of SWC is not only significantly correlated to good prognosis of full-term neonates with hypoxic-ischemic encephalopathy [10], but also can help predict neurological outcome in preterm children [11]. The aim of this study was to use aEEG to evaluate the effects of hyperbilirubinemia of full-term newborns on SWC. Our interest was focused not only on the occurrence number but also the structure of SWC and we tried to describe the detailed dose dependent effect of bilirubin exposure on SWC.

## Materials and Methods

### Patients

The study included all newborn babies with hyperbilirubinemia admitted to the Guangzhou Women and Children's Medical Center from January 2013 to December 2015. Hyperbilirubinemia was defined as the total serum bilirubin levels above the phototherapy limit recommended by American Academy of Pediatrics [12]. Specific inclusion criteria were gestational age 37–42 weeks; no history of phototherapy or exchange transfusion; no anti-convulsion drug history; no hypoxic-ischemic encephalopathy history and other disorders that may affect brain function or sleep. A general examination included a complete blood count, reticulocyte count, blood gas analysis, albumin levels, blood type, Coombs' test, blood culture, serum electrolytes, G-6-PD and head ultrasound. After examination, children with sepsis, intracranial hemorrhage and metabolic diseases were excluded. Total bilirubin (TSB) was measured by oxidizing method (Maccura, Sichuan, China) on the Hitachi 7600 autoanalyzer[13], and erythrocyte glucode-6-phosphate dehydrogenase deficiency was measured by quantitative G6PD/6PGD Ratio method (Micky, Guangzhou, China)[14].

These children were treated with phototherapy or exchange transfusion in hospital. The phototherapy and exchange transfusion standard was in accordance with the 2004 guidelines and updates of American Academy of Pediatrics [12].

#### aEEG settings

The methods and experimental procedures were similar to those described in our previous publications [15]. Briefly, bedside aEEG recordings were carried out shortly after the peak TSB was observed. Nicoletone Monitor (Thirty-two Channels video Monitor-one, VIASYS Healthcare, U.S.A.) was used in all recordings. All these procedures were conducted in an environmentally controlled room that was sound, light, temperature and humidity-proofed. Pre-recording preparation includes bath and removal of head fat tires. According to the international 10–20 system, prewired EEG caps with 9 recording electrodes modified for neonates were used for EEG recording. The nine recording leads were FP1, FP2, C3, CZ, C4, O1, O2, T3, T4. Patient ground and reference electrodes were placed in the frontal region. The wet electrodes used in the present study were the conventional sintered Ag/AgCl electrodes purchased from Suzhou Greentek (China). Each ‘wet’ electrode was filled with GT5 conductive gel (Greentek, Suzhou China) using a blunted needle and syringe. The impedance of the electrode was adjusted to the range of 100-5KΩ with the following recording parameters: time constant 0.5s, high-frequency filter 70Hz, and notch off. The aEEG was generated synchronously. Notable events (care operations) were marked by an EEG technician or nurse during the recording. The electrode impedance was kept in the required range. The recording was conducted for 12 hours for each patient.

The interpretation of EEG results was referenced to the current practice of clinical Electroencephalography[16].

The Ethical Committee of Guangzhou Women and Children's Medical Center approved this clinical study protocol (approval number 2012090839). A general informed consent as “All patients’ biological sample could be probably used for medical research, please contact the department of medical ethics to oppose. No objection shall be deemed to be the agreement” would be informed to all parents at admission. An electronic signed consent from the infants’ legal guardians was obtained after informed.

#### Data collection

**Fig 1:**
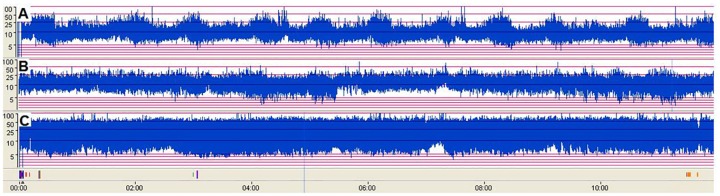
SWC on aEEG was recognized as periodic changes in bandwidth of the aEEG tracing. A: Normal SWC, more than ten consecutive changes in bandwidth are presented during a 12-hour aEEG tracing. B: Reduced SWC, four consecutive cycles. C: No SWC, no sinusoidal variations on aEEG background.

SWC on aEEG was recognized as periodic changes in bandwidth of the aEEG tracing [17]. The narrower parts (narrowband) of the aEEG trace corresponded to more continuous EEG background during wakefulness or active sleep (AS) periods while the broader parts (broadband) represented more discontinuous activity during quiet sleep (QS). Definition of normal SWC was made when at least 3 consecutive cycles were observed in aEEG tracing during a period of 5 hours, and the volatility fluctuation of the aEEG lower edge > 2μV[18]. In this study, we classified SWC by occurrence: ① Normal SWC: At least 7 consecutive cycles were present on aEEG tracing within12 hours (Fig 1A). ②Reduced SWC: Less than 7 consecutive cycles present during a 12-hour aEEG tracing(Fig 1B).③ No SWC: No sinusoidal variations of the aEEG background (Fig 1C).

The EEG record with the highest TSB level would be chosen for data analysis for each single patient. Data was collected from one of the bipolar channels (FP1-C3) on the aEEG recording. Data analysis was performed by two specific EEG analysts after removal of the artifacts. The following indices were obtained from each child: ①SWC occurrence number within 12-hour aEEG traces. ② Ratios of narrowband and broadband within 12-hour tracing. ③The duration of a single broadband from each child. ④The grades of EEG were documented. Normal EEG was marked as grade “1”, mild abnormal EEG as grade “2”, moderate abnormal EEG as “3” and severe abnormal EEG as “4”.

### Statistical analysis

Statistical analysis was performed using R 3.2.2 statistical package for windows (https://www.r-project.org/about.html). Variables with normal distribution were described as mean±standard error. Deviation and median (range) was used to describe abnormal distribution variables. Univariate comparisons of variables were made with Mann-Whitney U or Kruskal-Wallis tests for continuous variables and Fisher’s exact or χ^2^ tests for categorical variables. The association between TSB level and narrowband ratio, TSB level and broadband ratio were described by Spearman correlation analysis. Furthermore, a locally weighted scatter plot smoothing (LOESS) model was used to predict the number of SWC occurrence and the broadband ratio at the key levels of TSB. Comparison of single broadband period duration among groups was carried out by Kruskal Wallis Test. Wilcoxon rank sum test was used for further comparisons between groups. The level of significance was set at 0.05.

## Results

### Basic clinical characteristics

**Fig 2:**
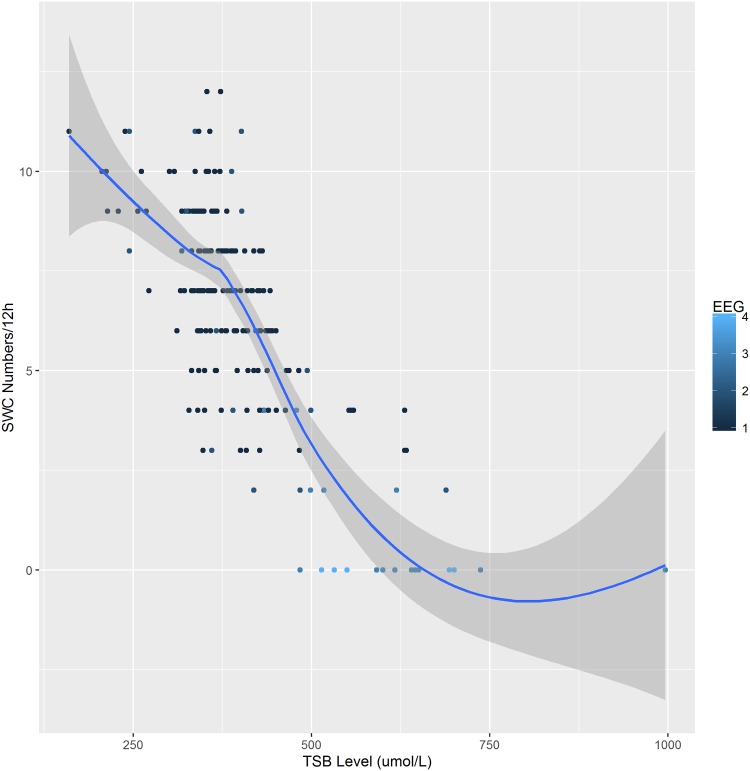
Negative correlation between TSB level and SWC occurrence numbers within 12 hours. The color shade of the scatterplot represented different levels of EEG abnormality grades from normal, mild abnormal, moderate abnormal to severe respectively.

A total of 203 cases aEEG results were carried out shortly as high TSB were observed in neonates. The range of TSB, gestational age (GA), postnatal age (PA), conceptional age (CA), birth weight (BW), weight gain after birth, weight at admission, gender, albumin (ALB), TSB/ALB, and diagnosis were summarized in Tab 1. Fourteen cases with hyperbilirubinemia (range from 483.9–996.2μmol/L) had no recognizable SWC on the aEEG record, 75 cases with reduced SWC (less than 7 cycles within 12 hours) could be seen from the TSB level range from 311.2 to 688.5μmol/L. The rest had normal SWC. The typical traces of the normal, reduced and no SWC were demonstrated in Fig 1A, 1B and 1C, respectively. The median occurrence declined gradually with elevated TSB levels *(r = -0*.*689*, *p = 0*.*000*,*Spearman's rank correlation*). Loess smooth model was applied to fit the nonlinear trend between the TSB levels and SWC occurrence (Fig 2). The color of the scatterplots referred to normal to severe abnormal EEG grades.

As shown in Table 1, TSB level of the subgroups of normal, reduced and no SWC indicated significant difference with each of the other two groups respectively (all P<0.001). So did the change of TSB/ALB, while the albumin level shown no difference between groups. The birth weight of both the normal and reduced group were significantly larger than that of the no SWC group (all P<0.001).

**Table 1:** Basic clinical characteristics.

	Total(n = 203)	Normal SWC(n = 114)	Reduced SWC(n = 75)	No SWC (*n* = 14)	*P*
TSB,μmol/L,median (range)	373.7(159.9–996.2)	353 (159.9–441.9) ***①	421.8(311.22–688.5) ***②	628.5(483.9–996.2) ***③	0.000
GA, d,median (range)	270(235–290)	271(239–290)	272(235–287)	264(251–285)	0.049
PA, d, median (range)	8.4 (0–36)	7(0–36)	7(0–22)	7 (1–19)	0.356
CA, d, median (range)	279.5 (245–311)	280(251–311)	279(245–300)	270(265–292)	0.062
BW, g, median (range)	3100(2100–4650)	3110 (2370–4650)***④	3190 (2100–4030)***④	2800 (2500–3200)	0.009
Gender m/f, n	99/104	56/58	34/41	9/5	0.425
ALB, g/L, median (range)	38.6(29.7–43.8)	38.9(34.9–42.3)	38.4(35.1–42.4)	38.25(29.7–43.8)	0.796
TSB/ALB, median (range)	9.2 (6.5–18.7)	8.6(6.5–9.4) ***①	12.7 (10.8–14.9)***②	16.6 (15.2–18.7)***③	0.000
Blood type incompatibility, n	66⑤	32	25	10	
ABO	21	13	7	1	0.828
Rh	1	1	0	0	0.675
G-6-PD deficiency	45	18	18	9	0.000

GA: gestational age, PA: postnatal age, CA: conceptional age, BW: birth weight. ALB: albumin. The P value at the last column indicated the comparison of the three subgroups (Kruskal-Wallis rank sum test or Pearson's Chi-squared test). P<0.05 represented the significant difference. Further comparison between groups are made by Wilcoxon rank sum test,

***P<0.001.

^①^:Compared to Reduced SWC group and No SWC group.

^②^:Compared to Normal SWC group and No SWC group.

^③^:Compared to Normal SWC group and Reduced SWC group.

^④^Compared to No SWC group.

^⑤^ABO incompatibility and G-6-PD deficiency co-existed on one patient.

We further analyzed 14 patients without recognizable SWC extensively (Clinical Profiles seen in Table 2, aEEG tracing seen in Fig 1C). Their TSB levels were >483.9μmol/L, predominantly male (9/14). They had symptoms of encephalopathy at admission, feeding difficulties; weight gain rates were low from birth to hospitalization. Causes were mainly G-6-PD deficiency (9/14), 4 cases had unclear cause and only 1 case had ABO incompatibility. In addition, 6 of the 9 cases who were undertaken for a MRI scan showed typical change of GP area (globipallidi) encephalopathy of hyperbilirubinemia. EEG and BAEP showed significant abnormalities. Two patients died during hospitalization, 4 patients were lost during following-up. Cerebral palsy was diagnosed in 2 patients, auditory pathway injury occurred in 4 cases of which 3 of them combined with mental retardation.

**Table 2:** Clinical Profiles of 14 Patients without SWC.

Patient number	Peak TSB (μmol/L)	Gender	Gestational age (d)	Onset Time(d)	Birth weight (g)	Diagnosis	MRI (yes/no)	Seizure (yes/no)	BAEP (grade)	EEG (grade)	Follow-up
1	996.2	M	40W	4	2850	G-6-PD deficiency	Ni	Y	3	3	CP
2	737.0	M	35W+6	4	3100	G-6-PD deficiency	Y	Y	5	3	MR, API
3	650.4	M	37W+5	3	3200	G-6-PD deficiency	Y	Y	5	3	LOST
4	645.3	F	38W	4	2980	G-6-PD deficiency	Ni	Y	5	3	Following-up
5	640.2	M	39W+2	1	2500	G-6-PD deficiency	Y	Y	5	3	LOST
6	616.9	M	40W+1	1	2800	G-6-PD deficiency	Y	Y	5	3	MR, API
7	600.0	M	40W+5	2	2700	G-6-PD deficiency	N	Y	3	3	LOST
8	591.2	F	38W+2	3	2790	BI	Y	Y	5	3	MR, API
9	483.9	F	37W+2	0	2700	unknown	N	Y	5	3	API
10	550.0	M	37W+1	3	3000	G-6-PD deficiency	Ni	N	Ni	4	DEAD
11	693.3	M	37W+2	3	3090	G-6-PD deficiency	Ni	N	Ni	4	DEAD
12	532.0	F	37W	3	2700	unknown	Y	Y	5	4	Following-up
13	700.0	F	37W+5	1	2670	unknown	N	Y	5	4	CP
14	514.2	M	37W+2	0	2580	unknown	Ni	N	4	4	LOST

BI: Blood group incompatibility; MRI: Typical MRI in GP area (GP, globi pallidi); T2WI, T2-weighted images, Ni, Not implemented; EEG, Grade 1–4 represented normal, mild abnormal, moderate abnormal and severe abnormal EEG respectively; BAEP, brain-stem auditory evoked potential; Grade 1–5 represented normal, mild abnormal, moderate abnormal, severe abnormal and no response respectively; CP, cerebral palsy; MR: mental retardation; API, Auditory pathway injury;

### With increasing levels of TSB, SWC structure also changed

**Fig 3:**
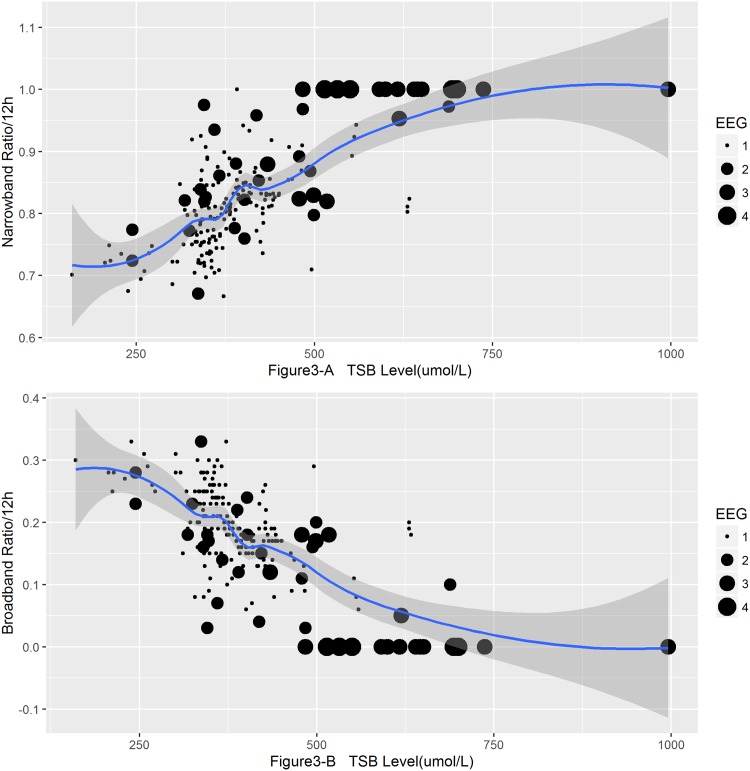
Scatterplot of increased TSB level with increased Narrowband ratio (A) and decreased Broadband ratio (B). Fitted and smoothed by LOWESS, span = 0.5, the different diameter of the scatterplots represented the grades of EEG abnormality.

SWC is composed of periodic changes of broadband and narrowband. We calculated the ratio of broadband and narrowband of each record in all identifiable SWC. The broadband ratio gradually decreased with the increasing levels of TSB (*r = -0*.*618*, *p* = 0.000), while the ratio of the narrowband gradually extended (*r = 0*.*618*, *p* = 0.000). Lowess smooth were applied to fit the nonlinear trend between the TSB levels and narrowband (Fig 3A) and broadband ratio (Fig 3B), respectively. The size of the scatterplots’ diameters provided us more information about the correlation between EEG abnormalities and TSB levels which indicated that the extremely hyperbilirubinemia did not always accompany by an abnormal EEG.

Since the SWC are composed of the narrowband and broadband (intermittent sleep are not in the realm of discussion in this paper), the ratio of narrowband length increase with the decrease of broadband ratio. The elevated TSB not only reduced the occurrence of SWC, but also changed its constituent.

### SWC occurrence and structure at different TSB levels could be predicted by a nonlinear model

**Fig 4:**
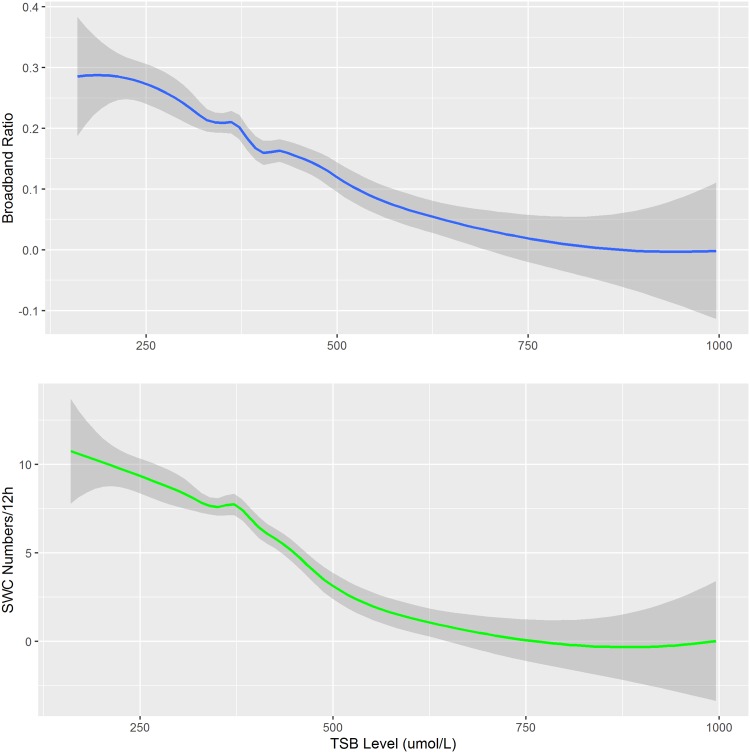
Loess model indicates the impact of TSB on SWC occurrence and Broadband ratio. The relationship of TSB with SWC occurrence and Broadband ratio were summarized in one picture. The two nonparametric regression curves with confidence belt indicated the predicted value of SWC occurrence and Broadband ratio respectively.

Loess model was used to predict the SWC occurrence number and broadband ratio at different key TSB levels (Table 3). We further summarized the data from Figs 2 and 3. As shown in Fig 4, the Loess predictive model clearly indicated the continuous impact of hyperbilirubinemia on the SWC, either occurrence number or structure.

**Table 3:** Predicted Broadband Ratio by Loess model.

Predicted values	TSB level(μmol/L)						
	200	300	400	500	600	700	800
Broadband Ratios(%)	28.75	24.58	15.66	11.72	5.93	2.36	0.09
SWC number(n)	10	8	6	3	2	1	0

Note: Predicted SWC numbers have been floored.

## Discussion

Significant neonatal hyperbilirubinemia can cause serious neurological damage, kernicterus, while low bilirubin exposure has only a subtle neurotoxicity. In our previous animal model study we have demonstrated [3] that an intravenous injection of indirect bilirubin after a certain time, under the condition that population spike is not affected, while the long term potentiation in the rat hippocampal CA3 area is suppressed. The results shows that neural plasticity characterized by long-term potentiation is different from the normal vulnerability of synaptic transmission to bilirubin. Since the syndrome contained in BIND is generally related to cognitive functions, damage to neural plasticity may be one of the pathogenesis of BIND. In addition, the brain damage caused by bilirubin can be expressed as an increase of central apnea [19] or swallowing dysfunction [20]. These changes are also caused by the less severe jaundice. The above evidences indicate that the assessment of bilirubin neurotoxicity requires long-term and comprehensive study.

Many studies have been reported on clinical observations of neurological outcome of hyperbilirubinemia with different degrees, but their conclusions were different. Like most other observations, we also used maximum TSB as an important criterion. However, this indicator did not cover many links through which bilirubin leads to nerve damage. These links affecting bilirubin neurotoxicity included duration of hyperbilirubinemia, serum albumin level, blood-brain barrier integrity, gestational age and the like. Thus, the results from observations in which TSB was an indicator were sometimes contradictory. For example, Newman et al[21] found completely normal neurological development at five years of age, in patients whose highest bilirubin at neonatal age was reported greater than 25mg/dl. In contrast, Soorani-Lunsing et al[22] reported that the bilirubin level of 13.6-26mg / dl in the neonatal period could cause mild nerve damage at 3–12 months. Because of the shorter observational period of the later study, these two results might suggest bilirubin neurotoxicity to a certain extent is reversible. However, these two studies were not carried out with electrophysiological observation and analysis for patients’ sleep. And, in case when positive signs of bilirubin neurotoxicity were found, no dose dependent effects were evaluated.

Sleep feature is an important means to measure the neurological developmental outcome of patients [23]. It has been proposed that circulating bilirubin serves as a photoreceptor and therefore plays an important role in sleep rhythm in adults [24]. Gürses et al.[8] used conventional EEG to observe infants’ sleep and found babies who experienced neonatal hyperbilirubinemia still showed sleep spindle abnormalities 12 weeks after birth. Our previous in-vivo study on rat models also showed that neural plasticity damage still occurred after [3] intravenously injected indirect bilirubin was removed from brain tissues. These results confirmed that bilirubin neurotoxicity may last for a long period of time.

Osredkar[18] used aEEG to observe the relationships between the SWC starting time, SWC quality and prognosis of full-term neonates with various degrees of asphyxia. Their observation perspective was the length of time in the process of SWC appearance in children with asphyxia. The median time of SWC starting times in mild, moderate, and severe asphyxia groups were 7, 33 and 62 hours after birth. They also looked at the SWC pattern, and normal SWC was significantly correlated with good prognosis. In accordance to this, our study also observed SWC of icteric patients through aEEG, but we studied the process of SWC disappearance and the relationship with different levels of TSB. We found TSB levels and the occurrence of SWC had a significant negative correlation, and we created a non-linear model to describe and predict the bilirubin exposed process. In fact the above two studies looked at two reverse processes of SWC, appearance and disappearance, respectively. Although both studied SWC change on aEEG, the specific method was not replaceable. This is because observed objects had different causes. Hypoxia at birth led to the delayed appearance of the physiological SWC, and the delayed time length had a positive change in the trend with the degree of brain damage. The present study showed that with increasing levels of TSB, the already existing SWC in icteric newborn gradually disappeared or even completely disappeared, and there was a reversed changing trend. Although the two studies had different starting points, the conclusion was consistent: the severity of the brain damage and SWC change was significantly correlated; the more serious of brain damage, the more significant of SWC variation. The extent of brain damage could affect the time of SWC's appears, SWC quantity and quality, and this effect did not have a cause specificity. In turn, we can use SWC start time, quality, quantity, and structure as important indicators to determine the severity and prognosis of brain damage.

Although the occurrence and structure of SWC has been studied under various disease conditions, a predictive model has never been made via a non-linear mathematic model. Some researchers used to split patients by different bilirubin levels and observed the dose dependent effects of bilirubin. On contrary, we analyzed the icteric newborns as a whole. We believed biologically reasonable hypothesis of our non-linear findings might be that effects of hyperbilirubinemia on SWC could be continuous, at least up to the point where SWC disappears. More observations could facilitate the accuracy of the above non-linear predictive model.

Furthermore in current study we found that 14 newborns with severe hyperbilirubinemia completely lost their SWC and the similar results were rarely reported previously. In 2005 a Canada team described 12 newborns with the TSB range 405–825μmol/L. Five cases of which had EEG abnormality including multifocal and generalized spikes and polyspikes, and discontinuous and intermittently asynchronous backgrounds [25]. Unfortunately SWC information has not been observed. The main cause of the severe neonatal hyperbilirubinemia in our study is G-6-PD deficiency (9 in 14 cases). The morbidity is higher than the data of the Canadian Paediatric Surveillance Program from 2002 to 2004 (20 in 93 cases) [26]. The reason might be the incidence of G-6-PD deficiency varied largely depending on the specific geographic region [27].

Animal model study showed the vulnerable region of bilirubin encephalopathy are cerebellum, the cochlea, the oculomotor nucleus, basal ganglia and hippocampus [28]. Hypothalamus—globus pallidus loop is closely related to the electrical activity of cortical neurons, and they both participate in the maintenance of the sleep-wake cycle[29]. Thus, the EEG activity change in acute phase of bilirubin encephalopathy has its corresponding neuropathological basis. Our research showed that SWC occurrence of hyperbilirubinemia newborn and TSB levels had a significant negative correlation. We observed abnormalities of sleep-wake cycle in patients with hyperbilirubinemia, but this cannot achieve the localization diagnosis of nerve injury. Because normal SWC requires the maturity and integrity of nervous system [30], any aspect of damage to the integrity can lead to abnormal SWC. From this point of view, although SWC cannot provide information on location, it may have a high sensitivity to subtle nerve damages with different degrees of exposure. Recently, Horst et al[31] also used aEEG to study preterm children (26–31 6/7 weeks gestational age) suffering from hyperbilirubinemia (TSB 124–291 μ mol/l), but did not find an association between SWC and TSB. The author believed this may be related to low bilirubin exposure.

We did not assess patients' long-term neurological prognosis, or the specific time of electrophysiological abnormalities and these are the drawback of present study. In reference to SWC findings of full-term HIE newborns, a poor prognosis can be determined from a lack of SWC [18,32], and for preterm children within 24 hours of birth, lack of SWC means brain damage [33]. Our research showed that the occurrence of SWC and TSB levels had a significant negative correlation particularly in a non-linear manner. With higher levels of TSB, the occurrence of SWC tended to decrease, manifested as the appearance of abnormal SWC. When TSB reached a certain level, SWC could disappear. In this study, SWC was not identified in 14 cases of children with TSB above than 483.9 μmol/L further confirming TSB’s role in brain injury. Therefore, we speculated that the SWC decrease may be one of the major characteristics of adverse neurological outcome in hyperbilirubinemia patients. With the information provided via the Loess model, the damage extent caused by hyperbilirubinemia could be intuitively displayed as the change of SWC occurrence and structure and this encourages the clinicians to take an active treatment.

In conclusions, the present study found that neonatal hyperbilirubinemia influences both the occurrence and structure of SWC. The change of SWC is a continuous phenomenon and could be described via a non-linear model. Further investigation should be done as further investigation could enrich the power of the non-linear predictive model.

## Supporting Information

S1 Table Clinical data. (XLSX)

S2 Table Clinical Profiles of 14 Patients without SWC. (XLSX)

S3 Table Original data of all SWC information. (XLSX)
